# RANDOMIZED CONTROLLED TRIAL OF LAY-DELIVERED INTERVENTION FOR OLDER ADULTS DISCHARGED FROM THE HOSPITAL

**DOI:** 10.1093/geroni/igad104.1343

**Published:** 2023-12-21

**Authors:** Kyaien Conner, Stephanie Kip, Amber Gum

**Affiliations:** University of Pittsburgh , Pittsburgh, Pennsylvania, United States; University of South Florida, Tampa, Florida, United States; University of South Florida, Tampa, Florida, United States

## Abstract

The second speaker will describe the background, aims, methods, and progress of a PCORI-funded RCT to reduce rehospitalization for Black/African American and Hispanic/Latino older adults after medical hospitalization. Across three regional hospitals, 402 older adults living with chronic illness (e.g., cardiovascular disease, diabetes, hypertension etc.,) will be enrolled while inpatient at one of our three partnering hospitals and randomized to receive one of three interventions in this comparative effectiveness trial: (1) the evidence-based Care Transitions Intervention (CTI), (2) CTI plus a peer educator intervention (CTI+Peer), or (3) usual care. Participants are assessed at 4 timepoints (baseline, 30 days, 90 days and 6 months) to examine differences in the primary outcomes of 30-day all cause hospital readmissions and emergency department visits. Secondary outcomes including quality of life, self-efficacy managing chronic disease, functioning, perceived social support and mortality. CTI Coaches and Peer educators participate in rigorous trainings which includes both didactic and interactive components to ensure interventionists are able to deliver the intervention with fidelity to the models. After training, CTI Coaches and Peer Educators participate in regular supervision meetings. They receive weekly virtual supervision check-ins from USF study staff and participate in a monthly group-based supervision in person. This trial involves close collaboration with leaders and coordinators at each hospital and an Area Agency on Aging (AAA) for recruitment, engagement of study interventionists and study dissemination. Across sites, 18 CTI Coaches and 11 Peer Educators have been trained and certified, and 145 participants have been enrolled (as of 3/8/2023).

